# CO-RADS versus CT-SS scores in predicting severe COVID-19 patients: retrospective comparative study

**DOI:** 10.1186/s43168-021-00060-3

**Published:** 2021-02-27

**Authors:** Niveen E. Zayed, Manar A. Bessar, Samah Lutfy

**Affiliations:** 1 Chest Department, Faculty of Medicine, Zagazig University, Zagazig, Egypt; 2 Radiology Department, Faculty of Medicine, Zagazig University, Zagazig, Egypt

**Keywords:** Chest CT, CT-SS, CO-RADS, COVID-19

## Abstract

**Background:**

The role of CT in assessing and plotting viral pulmonary affection land marking is its potential among other investigation tools, and the aim of the study was to compare the ability of two different CT-based scoring systems in discriminating severe COVID-19 disease.

**Results:**

Retrospective comparative study included 142 confirmed COVID-19 patients by real-time polymerase chain reaction (RT-PCR) test, with different degrees of disease (mild to severe), the data of patients collected from medical records, and patients with their first CT chest read for calculating CO-RADS and severity scoring system (CT-SS) score. The patients with severe COVID-19 disease were significantly older and had different comorbidities. The level of C-reactive protein, ESR, ferritin, and LDH were significantly higher in severe disease, *P* < 0.001. The ability of CT chest and its score bases (CT-SS and CO-RADS) were accurate in differentiation between mild/moderate and severe disease; AUC were 89% and 97%, respectively. The cutoff value of less than 7.5 and 4.5 for CT-SS and CO-RADS, respectively, can rule out severe COVID-19 by 90% and 97%, respectively.

**Conclusions:**

CT chest play a segregate role in COVID-19 disease, add on an advantage in clinical data in triage, and highlight the decision of hospital admission.

## Background

In December 2019, a pneumonia outbreak was reported by the International Committee on Taxonomy of Viruses in China as a result of a new zoonotic virus; the novel virus was named severe respiratory distress syndrome corona virus 2 (SARS-Cov-2) and can spread from human to another [1]. The WHO declared the disease as a pandemic disease by January 2020. The disease spread fast and globally; therefore, the need for rapid and accurate methods for early recognition and diagnosis of affected patients was increased [2].

The clinical features of SARS-Cov-2 infection and its preceding *beta corona* virus infections have been noted, and hence, most patients presented with influenza-like symptoms as fever, cough, and fatigue [3]. The disease may deteriorate causing severe respiratory distress syndrome as a consequence of pneumonia [4].

The gold standard method for diagnosing SARS-Cov-2 virus infection is real-time reverse transcriptase polymerase chain reaction (RT RT-PCT) or next-generation sequencing [3]. However, the sensitivity of these tests ranged from 42 to 83% depending on many factors like viral load, test sample quality, and duration of symptoms [5–9]. Moreover, the test did not impose the severity of the disease or its consequence [6]. Accordingly, another investigation side-by-side to clinical data will give good severity stratification and stage classification of patients.

Radiological evaluation of patients with SARS-Cov-2 infection particularly by chest computed tomography (CT) has a reported high sensitivity and enhances the clinical decision that is based on the degree of lung affection [6, 10]. Yang and his colleagues [11] introduce a severity scoring system (CT-SS) that depends on the degree of lung affection in chest CT and is recommended to be used for quick assessment of pulmonary affection. Moreover, in March 2020, the Dutch Radiological Society developed another score system based on chest CT and patient’s data; the COVID-19 Reporting and Data System (CO-RADS) included data of clinical finding and laboratory test results in addition to CT records [12]. The degree of suspicion ranged from very low to very high (CO-RADS categories 1–5), while category 0 reflects negative infection and category 6 establishes RT-PCR-positive SARS-Cov-2 infection at time of examination [12].

The current work is designed for enlightening the diagnostic utility of CT-SS in comparison with CO-RADS for evaluating patients with severe COVID-19.

## Methods

### Study design and patient grouping

A single-center retrospective comparative study included all RT-PCR positive cases for SARS-Cov-2 from the March 2020 to the end of June 2020 admitted in Saudi National Hospital. The study was approved by the Ethics Committee of Saudi National Hospital. A written informed consent was obtained from all participants

### Exclusion criteria

The study includes all patients referred to the hospital who were proved to have COVID-19 by RT-PCR throat swab. Pregnant women were excluded due to risk of CT, and also, patients with tuberculosis, interstitial lung diseases, and pulmonary malignancy were excluded to avoid interference with radiological presentation of COVID-19.

The positive PCR COVID-19 cases classified with regard to the level of disease severity: Mild disease (*n* = 22) included all cases with clinical symptoms and no changes in CT chest. Moderate cases (*n* = 62) involved all cases with respiratory symptoms with changes in CT. Severe cases (*n* = 17) defined by the presence of the following criteria: (1) respiratory distress, RR ≥ 30 beats/min, (2) resting blood oxygen saturation ≤ 93%, or (3) partial pressure of arterial blood oxygen (PaO2)/fraction of inspired oxygen (FiO2) concentration ≤ 300 mmHg. Critical ill cases (*n* = 41) included all severe cases that were deteriorating due to (1) respiratory failure and need of mechanical ventilation, (2) shock, and (3) other organ failure needing ICU monitoring treatment [13].

For the purposes of this study, mild and moderate cases were included in the same category (*n* = 84), while severe and critically ill cases were merged together (*n* = 58)

### Clinical workflow and disease evaluation

The data was collected from medical records and included demographic characteristic, clinical presentation, and routine laboratory investigation as CBC with lymphocytic count, C-reactive protein (CRP), erythrocyte sedimentation rate (ESR), d-dimer, lactate dehydrogenase (LDH), and arterial blood gas (ABG). In addition, the follow-up data included duration until conversion from positive to negative swab for SARS-Cov-2, length of stay in hospital, and mortality.

### Radiological work up

- Chest X-ray

The report of chest X-ray includes the site of lesion, presence of reticular, nodular, or opacity pattern.

b. CT protocol and reading

No specific protocol for CT imaging was applied; the study was retrospective, and two different multidetector CT scanners (Somatom Sensation 16 and Somatom Sensation 64; Siemens Healthineers) were used for all examinations as regards the manufacturer’s standard recommended for scanning parameters used in thoracic radiology. All images were reconstructed on workstation using multiplanar reformatting (MPR) technique. Two different radiologists, blinded to the patient’s clinical data, did the reading of each CT film; the least experience time on radiology filed for each collaborator was 10 years.

### Chest CT severity score assessment

*Yang* and his colleagues [11] developed a scoring system (CT-SS) that depended on opacification degree in the lung. The score was a modern adaptation of a previous method that was used in patients with SARS-Cov-1 [14].

Regarding the lung anatomical structures, all 18 lung segments were subdivided into 20 regions, which were then evaluated subjectively using scoring grades from 0 to 2; hence, 0 refereed to no involvement, while 1 and 2 represent less than and more than 50% involvement, respectively. The summation of individual’s scores of 20 regions pointed to total CT-SS score, which ranged from 0 to 40 points. The radiological terms that were established in use according to the Fleischner Society [15] includes ground glass opacity (GGO), crazy paving pattern, and pulmonary consolidation.

### CO-RADS score evaluation

The radiologist who observed the patient’s CT were familiar with CO-RADS score [12] from clinical experience on reading more than 45 CT chest; they used a drop list option tools in recording the points irrespective of the data from the recruited patients in the study

### Statistical analysis

The data were collected in Excel sheet and statistically analyzed using SPSS 22.0 for windows (SPSS Inc., Chicago, IL, USA). Continuous data was represented as mean and standard deviation (SD) and categorical data as number and percentage (%). The data normality has been checked using Shapiro Wilk test. Independent *t* test was used to compare between two different means, and chi-square test was used to compare the frequency of two groups or more. The accuracy of CO-RADS and CT-SS in diagnosing severe COVID-19 were assessed using receiver operating characteristic curve (ROC); the assumption was that the area under the curve (AUC) of 0.9 was significant, with a margin of error about 0.05 and 0.1 for type I and II errors, respectively. The minimum total calculated sample size was 70, and that for severe COVID-19 was about 35 using MedCalc 13 for windows (MedCalc Software bvba, Ostend, Belgium). All tests were two sided; *P* considered significant if < 0.05.

## Results

### Demographic characteristics of COVID-19 patients

About 142 patients had confirmed PCR for SARS-Cov-2, 84 of them presented by mild to moderate degree of disease severity. The severe COVID-19 cases were older in age and accompanied with multiple comorbidities (DM, HTN, and IHD) than mild/moderate one, *P* < 0.001, 0.003, and 0.01, respectively (Table 1). In the severe disease group, the mean (SD) duration before admission was 4.38 (1.61) days, while in the mild/moderate group, the duration was 3.67 (1) days, *P* = 0.008 (Table 1).

**Table 1:** Demographic data of COVID-19 patients

Factors	Mild/moderate (*n* = 84)		Severe/critically ill (*n* = 58)		*P*
Demographic characteristics	Mean/***N***	SD/%	Mean/***N***	SD/%	
Age	**35.4**	**9.71**	**47.4**	**10.6**	**< 0.001**^**§**^
Sex (male)	**70**	**83.33**	**46**	**79.31**	**0.61**
Smoking (yes)	**22**	**26.19**	**19**	**32.76**	**0.47**
Comorbidity (yes)					
DM	**4**	**4.76**	**20**	**34.48**	**< 0.001**^**#**^
HTN	**8**	**9.52**	**20**	**34.48**	**0.003**^**#**^
IHD	**0**	**0**	**8**	**13.79**	**0.01**^**#**^
Asthma	**4**	**4.76**	**8**	**13.79**	**0.12**
COPD	**0**	**0**	**2**	**3.45**	**0.51**
Hyperlipidemia	**4**	**4.76**	**2**	**3.45**	**0.74**
Clinical characteristic					
Exposure history (yes)	**28**	**33.33**	**17**	**29.31**	**0.66**
Duration from exposure till admission (days)	**9.14**	**1.96**	**9.18**	**1.98**	**0.96**
Duration before admission (days)	**3.67**	**1**	**4.38**	**1.61**	**0.008**^**§**^
Symptoms (yes)					
Fever	**76**	**90.48**	**52**	**89.66**	**0.89**
Cough	**64**	**76.19**	**54**	**93.1**	**0.01**^**#**^
Expectoration	**8**	**9.52**	**4**	**6.9**	**0.63**
Dyspnea	**12**	**14.29**	**38**	**65.52**	**< 0.001**^**#**^
Myalgia	**32**	**38.1**	**34**	**58.62**	**0.04**^**#**^
Sore throat	**28**	**33.33**	**21**	**36.21**	**0.76**
Hemoptysis	**0**	**0**	**5**	**8.62**	**0.051**
Diarrhea	**20**	**23.81**	**38**	**65.52**	**< 0.001**^**#**^
Loss of smell	**22**	**26.19**	**21**	**36.21**	**0.28**
Anorexia	**20**	**23.81**	**14**	**24.14**	**0.97**
Lab data					
TLC (10^9^/L)	**5.89**	**1.99**	**6.24**	**2.43**	**0.42**
HB (g/dL)	**15.36**	**1.52**	**15.03**	**2.01**	**0.35**
PLT (10^9^/L)	**194.1**	**53**	**198.5**	**71.2**	**0.72**
MPV (pg/L)	**10.26**	**2**	**11.53**	**1.65**	**0.001**^**§**^
L (%)	**27.08**	**7.51**	**19.92**	**7.26**	**< 0.001**^**§**^
ESR (mm/h)	**31.7**	**18.4**	**49.5**	**19.3**	**< 0.001**^**§**^
CRP (mg/L)	**39.5**	**23**	**93.4**	**50.3**	**< 0.001**^**§**^
Ferritin (ng/mL)	**297**	**171**	**738**	**426**	**< 0.001**^**§**^
LDH (U/L)	**340**	**154**	**496**	**234**	**< 0.001**^**§**^
AST (U/L)	**44.2**	**20.3**	**61.6**	**31.3**	**0.001**^**§**^
ALT (U/L)	**40**	**10.5**	**56.9**	**30.8**	**< 0.001**^**§**^
Creatine (mg/dL)	**0.75**	**0.144**	**0.924**	**0.228**	**< 0.001**^**§**^
d-dimer (positive)	**0**	**0**	**34**	**58.62**	**< 0.001**^**#**^
CPK-MB (IU/L)	**14.07**	**3.58**	**20**	**5.71**	**< 0.001**^**§**^
PO2 (mmHg)	**77.98**	**6.22**	**59.93**	**7.91**	**< 0.001**^**§**^
PCO2 (mmHg)	**38.95**	**2.32**	**34.77**	**4.03**	**< 0.001**^**§**^
PH	**7.39**	**0.02**	**7.36**	**0.40**	**0.58**
HCO3 (mEq/L)	**21.31**	**1.72**	**20.46**	**2.4**	**0.04**^**§**^
K (mmol/L)	**3.536**	**0.413**	**3.367**	**0.5**	**0.06**
Na (mEq/L)	**132.14**	**4.18**	**131.09**	**4.88**	**0.24**
CO-RAD	**2.33**	**1.18**	**4.86**	**0.39**	**< 0.001**^**§**^

^§^Independent *t* test

^#^Chi-square test, *P* considered significant if *P* < 0.05

Continuous data represented as mean and standard deviation (SD), and categorical data as number and percentage (%)

### Clinical and laboratory characteristics of COVID-19 patients

Considering the presenting symptoms, cough, dyspnea, and diarrhea were significantly associated with severe disease, *P* = 0.01, < 0.001, and < 0.001, respectively. Almost all laboratory markers (ESR, CRP, ferritin, LDH, and CPK-MB) were significantly higher in severe disease, as shown in (Table 1), *P* < 0.001 for all, while the lymphocyte % was significantly lower (19.9% mean as opposed to 27%), *P* < 0.001. Moreover, the positive d-dimmer was present in 58.62% of severe case, *P* < 0.001.

The CO-RAD score was significantly higher in severe case than in mild/moderate one; thus, the mean CO-RAD was 5 as opposed to 2 in other groups, *P* < 0.001.

### Radiological characteristics of COVID-19 patients

**Fig. 1:**
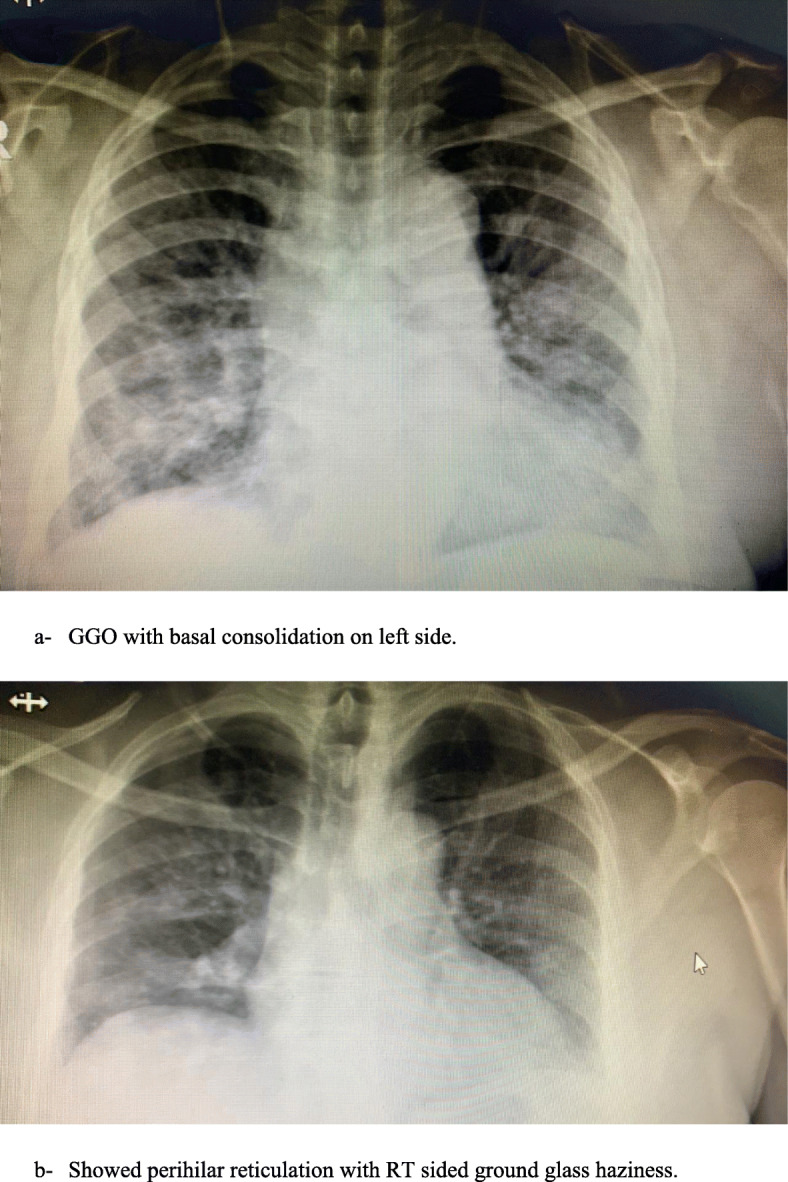
X-ray of COVID-19 cases. **a** GGO with basal consolidation on the left side. **b** Perihilar reticulation with RT-sided ground glass haziness

**Fig. 2:**
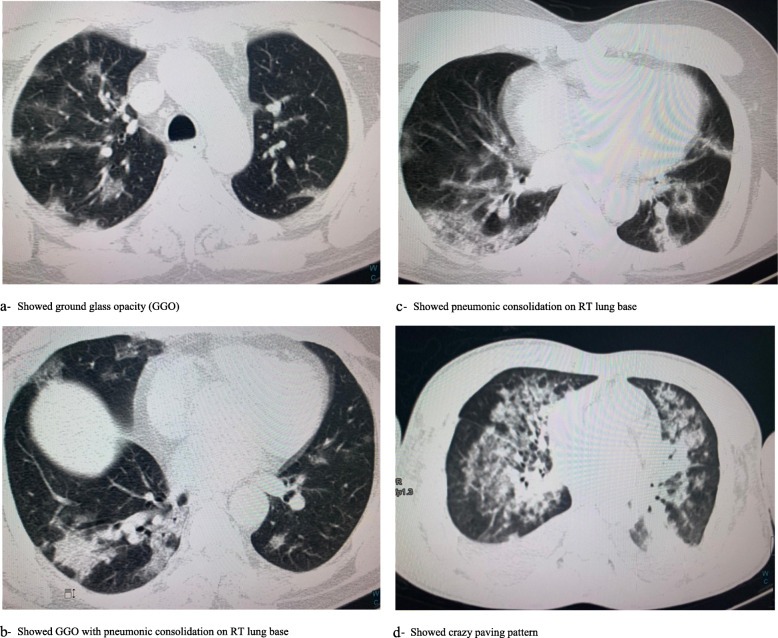
HRCT features of COVID-19 disease. **a** Ground glass opacity (GGO). **b** GGO with pneumonic consolidation on RT lung base. **c** Pneumonic consolidation on RT lung base. **d** Crazy paving pattern

Unilateral X-ray abnormality was significantly characterizing the mild/moderate disease, while almost all severe form had bilateral lesion, *P* < 0.001 (Table 2). As shown in Fig. 1a, bilateral peribronchial cuffing was reported, while Fig. 1b shows perihilar reticulation and haziness in RT lung base. The common features in CT chest were ground glass opacity with and without pneumonic consolidation as shown in Fig. 2.

**Table 2:** Radiological characteristic of COVID-19 patients

Factors	Mild/moderate (*n* = 84)		Severe/critically ill (*n* = 58)		*P*
	N	%	N	%	
X-ray abnormality	**36**	**42.86**	**58**	**100**	**< 0.001**^**#**^
Side					
Unilateral	**30**	**35.71**	**7**	**12.07**	**< 0.001**^**#**^
Bilateral	**6**	**7.14**	**51**	**87.93**	**< 0.001**^**#**^
Reticular shadow	**26**	**30.95**	**30**	**51.72**	**0.03**^**#**^
Opacity	**6**	**7.14**	**25**	**43.1**	**< 0.001**^**#**^
Nodules	**2**	**2.38**	**5**	**8.62**	**0.19**
CT changes					
Anterior segment (L)	**0**	**0**	**25**	**43.1**	**< 0.001**^**#**^
Apical segment (L)	**0**	**0**	**17**	**29.31**	**< 0.001**^**#**^
Posterior segment (L)	**0**	**0**	**23**	**39.66**	**< 0.001**^**#**^
Superior lingual segments (L)	**12**	**14.29**	**31**	**53.44**	**< 0.001**^**#**^
Inferior lingual segments (L)	**12**	**14.29**	**32**	**55.17**	**< 0.001**^**#**^
Superior segments (L)	**8**	**9.52**	**29**	**50**	**< 0.001**^**#**^
Anterior basal (L)	**12**	**14.29**	**32**	**55.17**	**< 0.001**^**#**^
Medial basal (L)	**10**	**11.9**	**30**	**51.72**	**< 0.001**^**#**^
Lateral basal (L)	**10**	**11.9**	**35**	**60.34**	**< 0.001**^**#**^
Posterior basal (L)	**8**	**9.52**	**39**	**67.24**	**< 0.001**^**#**^
Anterior segment (R)	**2**	**2.38**	**23**	**39.66**	**< 0.001**^**#**^
Apical segment (R)	**2**	**2.38**	**16**	**27.59**	**< 0.001**^**#**^
Posterior segment (R)	**4**	**4.76**	**25**	**43.1**	**< 0.001**^**#**^
Medial (R)	**8**	**9.52**	**32**	**55.17**	**< 0.001**^**#**^
Lateral (R)	**10**	**11.9**	**34**	**58.62**	**< 0.001**^**#**^
Superior (R)	**4**	**4.76**	**30**	**51.72**	**< 0.001**^**#**^
Anterior basal (R)	**16**	**19.05**	**38**	**65.51**	**< 0.001**^**#**^
Medial basal (R)	**22**	**26.19**	**37**	**63.79**	**< 0.001**^**#**^
Lateral basal (R)	**24**	**28.57**	**37**	**63.79**	**0.001**^**#**^
Posterior basal (R)	**26**	**30.95**	**32**	**55.17**	**0.016**^**#**^

^#^Chi-square test, *P* considered significant if *P* < 0.05

Continuous data represented as mean and standard deviation (SD), and categorical data as number and percentage (%)

**Fig. 3:**
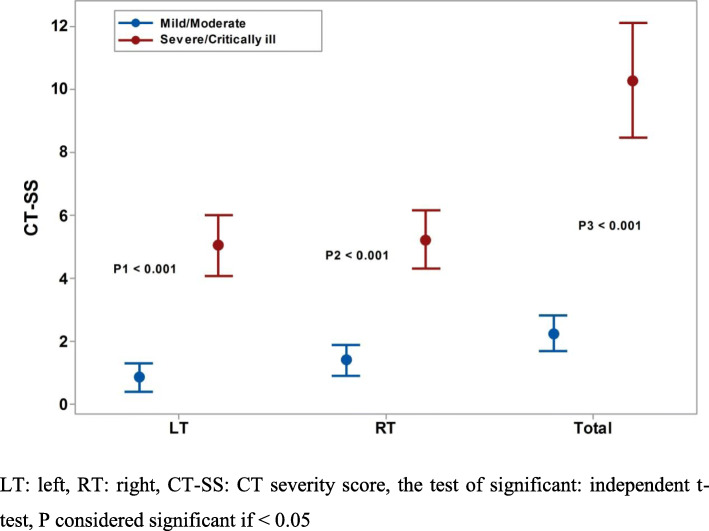
CT-SS score in different COVID-19 disease severity: LT, left; RT, right; CT-SS, CT severity score; the test of significance: independent *t* test, *P* considered significant if < 0.05

The total CT-SS score was significantly higher in severe disease as well as the score of left and right side; hence, the mean (SD) were 10 (7), 5 (4), and 5(4) respectively, as opposed to 2 (2), 1 (1), and 1(1), respectively, *P* < 0.001 for all (Fig. 3).

### Prognosis of COVID-19 patients

About 2/3 of severe cases were admitted in ICU (70.69%) and 1/3 need MV support and developed ARDS (29.31 and 37.39%, respectively). The conversion time (days) to negative swab and length of stay (LOS) in hospital were longer in severe cases than in mild/moderate cases, *P* < 0.001 for both (Table 3). Mortality was reported only in severe groups, as about 12% of them died (Table 3).

**Table 3:** Prognosis of COVID-19 cases

Factors	Mild/moderate (*n* = 84)		Severe/critically ill (*n* = 58)		*P*
	***N***/Mean	%/SD	***N***/Mean	%/SD	
ICU need	**0**	**0**	**41**	**70.69**	**< 0.001**^**#**^
MV	**0**	**0**	**17**	**29.31**	**< 0.001**^**#**^
ARDs	**0**	**0**	**22**	**37.93**	**< 0.001**^**#**^
Death	**0**	**0**	**7**	**12.07**	**< 0.001**^**#**^
Duration to conversion (days)	**6.5**	**1.19**	**9.38**	**2.7**	**< 0.001**^**§**^
LOS (day)	**6.76**	**1.25**	**8.69**	**2.01**	**< 0.001**^**§**^

^§^Independent *t* test

^#^Chi-square test, *P* considered significant if *P* < 0.05

Continues data represented as mean and standard deviation (SD), and categorical data as number and percentage (%)

### Performance of CT-SS and CO-RAD score in predicting severe COVID-19

**Fig. 4:**
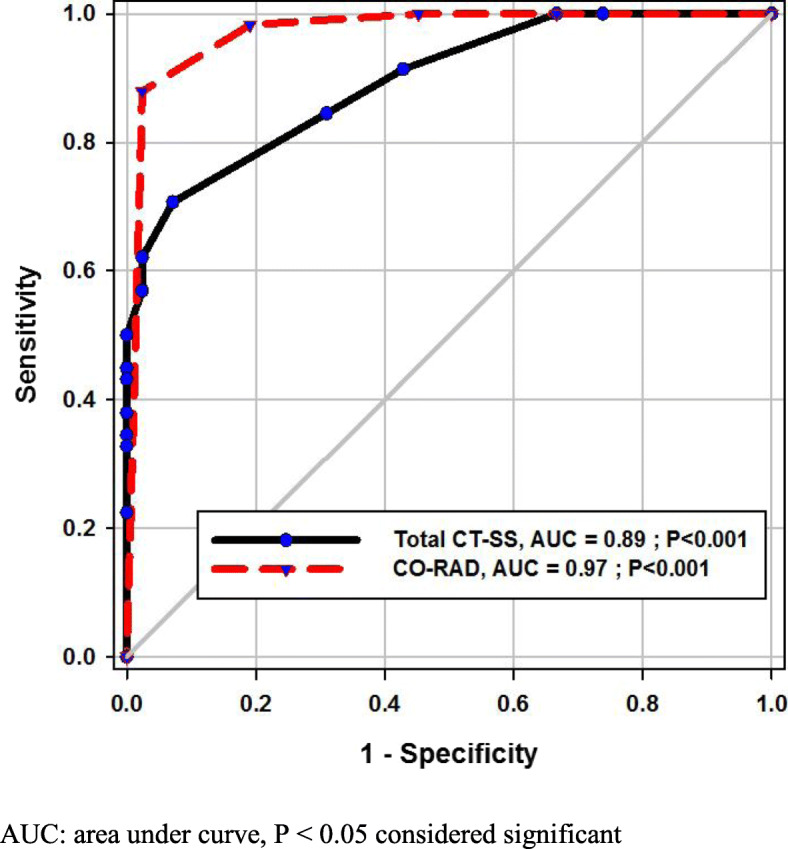
ROC curve of total CT-SS and CO-RADS score: AUC, area under the curve; *P* < 0.05 considered significant

Both CT-SS and CO-RAD score had excellent performance in predicting severe COVID-19; hence, the AUC were 0.89 and 0.97, respectively, *P* < 0.001 for both (Fig. 4). However, in comparing the capability of them, CO-RAD score had the upper hand, as the area difference was − 0.078, and *P* = 0.002 (Table 4).

**Table 4:** Validity of CT-SS score and CO-RAD score in prediction of severe COVID-19

Test variables	Total CT-SS	CO-RAD
ROC curve area	**0.89**	**0.97**
95% confidence interval	**0.8438 to 0.9460**	**0.9504 to 0.9955**
*P* value	**< 0.0001**	**< 0.0001**
Area difference	**− 0.07800**	
Standard error	**0.02976**	
95% confidence interval	**− 0.1277 to − 0.02832**	
*P* value	**0.002089**	

*ROC* Receiver operating characteristics, *P* < 0.05 considered significant

The sensitivity, specificity, positive predictive value (PPV), and negative predictive value (NPV) of CT-SS and CO-RAD score were different at different cutoff points, as shown in Table 5, the best cutoff point of CT-SS with higher sensitivity was > 1.5, while that with higher specificity, the cutoff point was > 7.5. Furthermore, in CO-RAD score, the specificity for severe COVID-19 was higher (98%) at cutoff point > 4.5, with acceptable sensitivity (88%).

**Table 5:** Performance of CT-SS and CO-RAD score in predicting severe COVID-19

Test	Sensitivity	95% CI	Specificity	95% CI	PPV	NPV
Total CT-SS						
Cutoff >						
1.5	**100%**	**0.9384 to 1.000**	**33%**	**0.2342 to 0.4446**	**25%**	**100%**
2.5	**91%**	**0.8102 to 0.9714**	**57%**	**0.4588 to 0.6789**	**32%**	**97%**
7.5	**50%**	**0.3658 to 0.6342**	**100%**	**0.9570 to 1.000**	**100%**	**90%**
CO-RAD						
Cutoff >						
2.5	**100%**	**0.9384 to 1.000**	**55%**	**0.4352 to 0.6566**	**33%**	**100%**
3.5	**98%**	**0.9076 to 0.9996**	**81%**	**0.7092 to 0.8870**	**53%**	**100%**
4.5	**88%**	**0.7670 to 0.9501**	**98%**	**0.9166 to 0.9971**	**89%**	**97%**

*CI* confidence interval, *PPV* positive predictive value, *NPV* negative predictive value

## Discussion

The chest CT became an essential diagnostic tool during the COVID-19 outbreak, especially the thin section CT image [16]. Like other viral pneumonia, the features of CT include ground glass opacification, segmental and sub-segmental thickness (crazy paving), consolidation, and interstitial infiltration [17, 18]. The typical findings in CT of COVID-19 patients were patchy, rounded segmental and sub-segmental ground glass opacification that may be deteriorated to consolidation [19, 20]. The predominant lesion distribution in the posterior and basal part in our study was closely matched with that of Song et al. [21] who found that 82% of COVID-19 patients had posterior lung involvement, as well as to Yang et al.’s [11], whose results added that the consolidation was significantly associated with disease severity too. Moreover, the data on SARS-Cov-1 and MERS Cov infections signify that posterior segment affection predominance [22, 23].

The role of chest X-ray in our study was limited and less sensitive, particularly in the early stage of the disease, as the abnormality was closely related to severe disease (100% versus 42.86% in mild/moderate disease), *P* < 0.001. On the other hand, the tiny opacity in any areas of 20 subjective CT locations was denoted and scored. Some authors accounted that the role of X-ray was established in the follow-up stage; therefore, the sensitivity of X-ray was 59% [24, 25].

In our study, the total CT-SS was significantly higher in severe COVID-19 disease than in the mild/moderate group; thus, the mean (SD) were 10 (7) as opposed to 2 (2), which was in agreement with Yang et al.’s study [11]. Additionally, at 2.5 point, the sensitivity was 91% that gave NPV about 97%; also, by increasing the threshold point to 7.5 or more, the specificity increased up to 100% with NPV about 91%. There was a discrepancy between our cutoff value and that of Yang et al.’s study [11], in which the chosen value was higher than our value of 19.5, giving sensitivity and specificity about 83.3% and 94%, respectively. It may be due to his small number of severe COVID-19 cases than that of ours (18 versus 58) even with equal performance of CT-SS in discriminating severe COVID-19 disease (AUC = 89%).

In a study conducted by Bellini et al. [26] to validate CO-RADS accuracy in diagnosing COVID-19 cases, the threshold value of 4 and more provided reasonable sensitivity and specificity of 61% and 81%, respectively, with AUC about 72%. While in our study, the same cutoff point has been used to predict severe COVID-19 cases and offered sensitivity and specificity about 88% and 98%, respectively, with AUC about 97%. Our results were quite close to those of Prokop et al.’s [11], who found the accuracy of CO-RADS was 91%. The difference in the results between Bellini and Prokop could be related to observer experience; thus, the latter involved an expert radiologist with at least 20 years of expertise in CT reading. So, the learning curve of the radiologist may be considered as a factor that implied the outcome. The results of the current work show that among the CT-based score systems of perceptive COVID-19 disease particularly in the severe stage, the CO-RADS score had significant insight over CT-SS, hence the AUC difference; 95% CI was (− 0.07; − 0.1277 to − 0.02832), *P* = 0.002. Furthermore, our study was the first of its kind to provide comparative analysis between two different radiological-based score systems (CT-SS and CO-RADS), as well as to use the aforementioned score in discriminating severe COVID-19 disease, not only positive RT-PCR based COVID-19. Both scores had excellent accuracy, 89% for CT-SS and 97% for CO-RADS. In spite of that, this retrospective study had some limitation: first, being a one-center study with limited numbers of cases and, second, the first CT of patients at presentation time was used for analysis, while the rest of follow-up CT and progressive data, which may be implicated with the degree of lesions, had been overlooked. Consequently, another complementary study was needed to find the variability between the initial CT presentations and the follow-up one.

## Conclusion

In conclusion, the present work highlights the important role of CT chest and its score base; CT-SS score of less than 7.5 and CO-RADS less than 4.5 could rule out severe COVID-19 disease by NPV about 90% and 97%, respectively. Moreover, CT score in addition to patient’s clinical parameters empowers the triage options especially during the peak of the pandemic wave.

## Data Availability

The database used and analyzed during the current study are available from the corresponding author on reasonable request

## Abbreviations

ABG: Arterial blood gas
ALT: Alanine aminotransferase
ARDS: Acute respiratory distress syndrome
AST: Aspartate transaminase
AUC: Area under the curve
CBC: Complete blood count
CI: Confidence interval
CO-RADS: COVID-19 Reporting and Data System
COPD: Chronic obstructive pulmonary disease
COVID-19: Corona virus disease 19
CPK-MB: Creatine phosphokinase myocardial band
CRP: C-reactive protein
CT: Computed tomography
CT-SS: Severity scoring system
DM: Diabetes mellitus
ESR: Erythrocyte sedimentation rate
FiO2: Fraction of inspired oxygen
GGO: Ground glass opacity
HB: Hemoglobin
HRCT: High-resolution computed tomography
HTN: Hypertension
ICU: Intensive care unit
IHD: Ischemic heart disease
LDH: Lactate dehydrogenase
LOS: Length of stay
MERS Cov: Middle East respiratory syndrome-related corona virus
MPR: Multiplanar reformatting technique
MPV: Mean platelet volume
MV: Mechanical ventilation
NPV: Negative predictive value
PaO2: Partial pressure of arterial blood oxygen
PLT: Platelet count
PPV: Positive predictive value
ROC: Receiver operating characteristic curve
RT RT-PCT: Real-time reverse transcriptase polymerase chain reaction
RT-PCR: Real-time polymerase chain reaction
SARS-Cov-2: Severe respiratory distress syndrome corona virus 2
SD: Standard deviation
TLC: Total leucocytes count
WHO: World health organization
